# Applying ecosystem services as a framework to analyze the effects of alternative bio-economy scenarios in Nordic catchments

**DOI:** 10.1007/s13280-020-01348-2

**Published:** 2020-06-27

**Authors:** Jan E. Vermaat, Bart Immerzeel, Eija Pouta, Artti Juutinen

**Affiliations:** 1 Faculty of Environmental Sciences and Natural Resource Management, Norwegian University of Life Sciences (NMBU-MINA), Ås, Norway; 2 Finnish Natural Resources Institute Finland (Luke), Helsinki, Finland; 3 Finnish Natural Resources Institute Finland (Luke), Oulu, Finland

**Keywords:** Cascade, Final ecosystem services, Land use change, Shared socio-economic pathways

## Abstract

The inherently unknown future development of a Nordic bio-economy was studied with four scenarios applied in an ecosystem service assessment framework. This framework couples CORINE land use cover with estimates of 15 final ecosystem services from the CICES 5.1 classification in biophysical and monetary terms. Current land use in two catchments, Lillebæk (83% cropland, area 4.7 km^2^, Denmark) and Ovre Haldenvassdraget (67% forest, 1006 km^2^, Norway) was compared with four scenarios for 2050. One scenario focusing on sustainability and environmental awareness led to considerable changes in land use and ecosystem service delivery (more diverse provisioning and higher value of regulating services, but not a higher total economic value), whereas the other three did not deviate markedly from the current scenario. Projected land use scenarios were verified with experts and stakeholder representatives. We conclude that the framework has sufficient resolution to show differences in service delivery among scenarios.

**Electronic supplementary material:**

The online version of this article (10.1007/s13280-020-01348-2) contains supplementary material, which is available to authorized users.

## Introduction

The transition of Nordic societies away from fossil fuel dependence towards a stronger dependence on and a more diversified use of bioresources, particularly those from forestry, is a move towards a state often referred to as ‘the bio-economy’ (Bugge et al. 2016; Eyvindson et al. 2018). Such a future ‘bio-economy’ is inherently uncertain and may proceed along widely contrasting trajectories. It is likely to have profound consequences for land use cover and the intensity of use, and thus for the multiple ways landscapes provide benefits to society (e.g., Nelson et al. 2009; Triviño et al. 2018). The inherent uncertainty of a future bio-economy or any trajectory of societal development is usefully charted with scenarios (Lorenzoni et al. 2000; Busch 2006; O’Neil et al. 2017). Scenarios have become a benchmark tool for projecting contrasting but plausible alternative pathways of development. We will only briefly describe the different scenarios we use for the potentially divergent ways in which a bio-economical change in natural resource exploitation can develop in Nordic landscapes because they are the subject of Rakovic et al. (2020).

Catchments are naturally bounded spatial units in the landscape that aggregate into larger river basins, which are the administrative spatial units for water management (Moss 2012). Often, these river basins match only imperfectly with administrative units, but they still share a scale where policy and management of water, agriculture, forestry, and biodiversity conservation have their interface, and where different sectors encounter conflicts of interest and have variable priorities. Spatial planning requires to resolve such conflicts; doing so at the catchment scale is particularly relevant for identifying spatial, for example, upstream–downstream, dependencies (Nelson et al. 2009; Bouma and Van Beukering 2015). It is thus at this larger scale where a comprehensive, cross-sectoral assessment has a high relevance for targeted policy development and evaluation. Multiple, possibly interactive, effects of changes in land use can be assessed comprehensively with ecosystem services as an analytical framework (e.g., Nelson et al. 2009), and this approach is increasingly adopted in policy development and land use decision-making (e.g., Guerry et al. 2015). The purpose of this paper is to explore the possibilities and limitations of the ecosystem services approach for such a catchment-scale assessment of the effects of a future bio-economy.

We will first briefly review the status of ecosystem service assessment methods, and then address the way changes in land use can affect the provision of multiple ecosystem services. We then develop our method from the cascade perspective of Mononen et al. (2016), hence our short name ‘Mononen-cascade.’ It attempts to integrate all potentially relevant services across a catchment or landscape, while it is minimal in its assumptions, allows implementation of scenarios, keeps track of the different services in a transparent way, and uses monetary values as a tangible, not absolute, measure for comparison of scenario outcomes. We subsequently apply this method for two Nordic catchments using the scenario articulations from Rakovic et al. (2020) and conclude with a discussion of the weaknesses and possibilities of the approach in assisting land use policy evaluation and bio-economy planning.

## Method: developing our analytical framework

### Ecosystem service assessment

Since the seminal Millennium Ecosystem Assessment (MEA 2005), the number of publications addressing the subject of ecosystem services in one way or another has grown exponentially (e.g., Fisher et al. 2009; Boerema et al. 2017). As a follow-up to the MEA, the TEEB exercise (the Economics of Ecosystems and Biodiversity, Kumar 2010) led to several national ecosystem service assessments (e.g., Watson and Albon 2011; Meyerhof et al. 2012; Bateman et al. 2013; Mononen et al. 2016), the development of a systematic classification of ecosystem services (CICES, Haines-Young and Potschin 2017), as well as the conceptualization of ecosystem services as a cascade (Haines-Young and Potschin 2010). Together with the CICES classification, the cascade conceptualization is acquiring a benchmark status for ecosystem services assessments in Europe (e.g., Boerema et al. 2017; La Notte et al. 2017). The ‘Mononen-cascade’ we use consists of four elements: ecosystem structure, ecosystem function, societal benefit, and societal value. Haines-Young and Potschin (2010) originally labeled the third step, societal benefit, as ‘service (flows),’ and the last as ‘benefit (value).’ Different authors tried to precisely pinpoint where in the cascade the true service ought to be located, for example, in-between function and benefit, or have added elements to the cascade. Both Mononen et al. (2016) and Boerema et al. (2017), however, argue that there is no conceptual reason for this as one can consider the whole cascade as the ecosystem service, since all elements are necessary, one could see the two directly linked flows function and benefit together forming the ecosystem service, or one can argue that it is the benefit that is finally of use to humans and thus is a final service (cf. Bateman et al. 2011).

### Requirements for a framework

An analytical framework that integrates all potentially relevant services across a landscape has the intrinsic risk to become overly complex in the interactions and feedbacks that are included and unbalanced in its degree of detail across entities of interest (e.g., Díaz et al. 2015). We argue therefore that such a framework should be (a) minimal in its assumptions (e.g., Mononen et al. 2016; Boerema et al. 2017), (b) linked to a simple and traceable classification of land (and water) cover and (c) allow to keep track of the different services in a transparent way. The latter implies a degree of consistency in detail among services. In order to be useful for decision-making it should also (d) allow for the implementation of scenarios to deal with variation, (e) allow for spatial disaggregation, and (f) be amenable for interaction with stakeholder representatives during its development. We will return to these six criteria in the discussion.

Finally, we posit that the estimation of monetary values for final services and an aggregation of these into an estimate of total economic value (TEV, as a rate per area and year) would work as a tangible indicator for comparative use in scenario evaluations and in communication with policy makers. The valuation step, in principle, is similar to a simplified weighing in multi-criteria analysis (MCA) (e.g., Wittmer et al. 2006), or the summing of a Likert-like ‘importance’-score ranging from 1 to 5, as applied in e.g., Burkhard et al. (2009). However, monetary valuation causes a differential weighing of the different services, rather than treating all individual services as equal.

### Structure of the framework

**Fig. 1:**
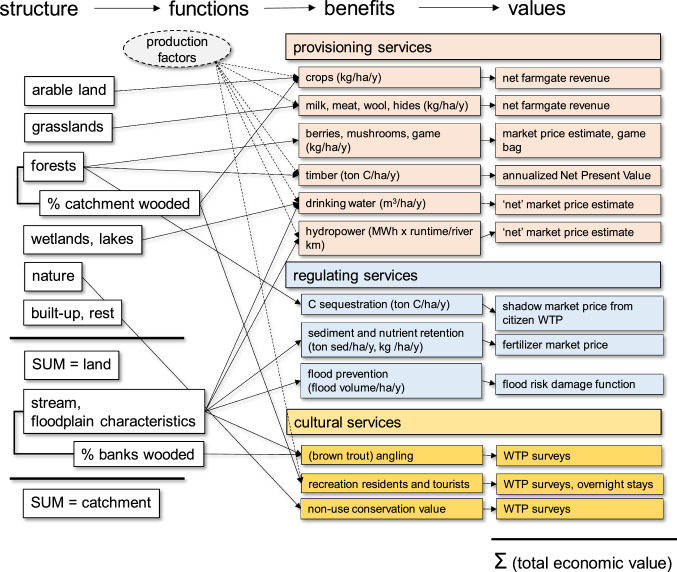
Flow of ecosystem services from ecosystem structure expressed as different types of land use, catchment, and river metrics to monetary value estimates of annual service flow for 15 different ecosystem services. Milk, meat wool, and hides as well as berries, mushrooms, and game are merged in this figure for clarity; also not all possible linkages are shown). Provisioning, regulating, and cultural services are indicated with different colors for clarity. The shaded factor ‘production factors’ is qualitatively illustrating that some services require substantial input before they are available for use, and not all this effort is included in, for example, net farmgate revenue. Further explanation in Table 1

We used the ‘Mononen-cascade’ (Mononen et al. 2016), and for each service identified indicators for ecosystem structure, biophysical service flow or benefit and societal (monetary) value (Fig. 1). We found that separate indicators for ecosystem function did not add much clarification and decided to keep these implicit. Several European authors have built an ecosystem service assessment upon the CORINE land cover classification because it is harmonized across national borders (e.g., Burkhard et al. 2009; Vermaat et al. 2016). We therefore used CORINE land cover as a simple benchmark indicator of ecosystem structure. From the CICES 5.1 classification (Haines-Young and Potschin 2017), we selected 15 independent final ecosystem services which we considered potentially relevant for our study catchments (Table 1). We interpret services as final when these are the final biophysical entities used or appreciated (cf. Bateman et al. 2011). We derived the service flow in biophysical units per area and year and then estimated a monetary value for each service using local data where possible, or alternatively taking European values from earlier work, the latter of course is a source of potential inaccuracy (Vermaat et al. 2016). All these are compiled in a simple spreadsheet model, and its general logic is depicted in Fig. 1. Monetary estimates are in euros but not value-standardized to a specific year. Monetary values are generally from the period 2005–2015, so can be regarded as approximate 2010 values. Also, since price levels and variability in the currency of the two studied countries are generally similar, we have not carried out a purchasing parity correction. The spreadsheet is available as Supplementary Material S1.

**Table 1:** Relevant ecosystem services selected and aggregated when necessary from CICES 5.1 and quantified in Lillebæk and Ovre Haldenvassdraget. Value estimates are expressed as euro per ha catchment per year, and monetary values can be considered approximately 2010 values. For simplicity, we have omitted the step from biophysical service flow (e.g., kg ha^−1^ year^−1^) to its monetary value estimate (€ ha^−1^ year^−1^) where it is a simple linear link

Service (CICES 5.1 codes)	Description	Explanation, sources
Provisioning		
Crops (1.1.1.1)	Net farmgate revenue arable farms (400 € ha^−1^ [cropland] year^−1^)	Not market price of product, but net farmers revenue before tax and subsidy, based on Mueller and Mueller (2017) from a standard set of representative and intensively monitored farms in Rheinland-Pfalz, Germany. Conditions will differ from those in Denmark and Norway, but we assume that net revenues will not grossly deviate for an order of magnitude comparison
Dairy, meat, hides, fleece (1.1.3.1 and 1.1.3.2)	Net farmgate revenue dairy farms (500 € ha^−1^ [grassland] year^−1^)	As for crops based on Mueller and Mueller (2017). We see dairy products as the final service, and not cattle fodder.
Timber (1.1.5.2)	Conservative annualized net present value estimate based on annual beech or fir productivity for Northern and Central Europe (200 € ha^−1^ [forest] year^−1^)	We use a conservative low-end value based on Duncker et al. (2012, different scenarios with different rates of interest, range of 0–800 € ha^−1^ year^−1^), Hastreiter (2017, 130 € ha^−1^ year^−1^, net revenue small-scale forestry), and Boesch et al. (2018, 300 € ha^−1^ year^−1^)
Berries and mushrooms, game (1.1.5.1 and 1.1.6.1)	Conservative estimate from a comparative European review, mainly Germany and France (24 € ha^−1^ [woodland] year^−1^, 80% due to game)	Adjusted from Schulp et al. (2014), which has a similar estimate as Boesch et al. (2018) report. Values for Denmark and Norway will differ from central Europe due to difference in game density, different species, and differences in the human population engaged in hunting and berry and mushroom collecting. This is therefore likely an underestimate
Hydropower (4.2.1.3)	Reported current locally generated hydropower (0-2 € ha^−1^ [whole catchment] year^−1^)	Values are normalized from length of higher order streams to catchment area. Consumer price is halved to reduce the benefits accumulating in the value chain and remain comparable with net farm gate revenues as for crops and dairy. In Lillebæk no hydropower is generated, but in Ovre Haldenvassdraget there is, at the very downstream end, at Ørje: 9 GWh year^−1^. We have taken half the median electricity price from Statistics Norway resulting in a value of 0.05 € kWh^−1^; There is no current production upstream of Ørje, but we estimate that another 4.5 GWh year^−1^ could be generated, leading to a potential value estimated of 89 kWh ha^−1^ year^−1^, or 2 € ha^−1^ year^−1^
Drinking water (4.2.1.1)	Reported local extraction and use of surface water (0–5 € ha^−1^ [whole catchment] year^−1^)	This may be river water infiltrated into aquifers and then extracted again, or direct use. Market price is halved to reduce the benefits accumulated in the value chain and remain comparable to net farm gate revenue. Values are normalized to the whole catchment area. In Lillebæk there is no reported drinking water produced from aquifer or stream, but in Halden this is the case: based on municipality reports 2700 m^3^ day^−1^ are used from the lakes directly in the stream system; the m^3^ consumer price is halved as explained, leading to a value of 0.5 € m^−3^, and the product is normalized to catchment area
Regulating		
Greenhouse gas reduction (2.2.6.1)	Carbon sequestration in coniferous and deciduous woodland and riparian bushes at, respectively, 6, 5, and 4 ton C ha^−1^ year^−1^ (based on Paul et al. 2009)	In all scenarios except NBP1 a low price of 5 euro per ton C is used Elsasser et al. (2010) and Loeschel et al. (2013). For NBP1 we assume a moderate increase due to the further development of a carbon credit market to 20 euro Vermaat et al. (2016) and Boesch et al. (2018
Erosion control: lateral sediment Retention (2.2.1.1 and 2.2.1.2)	Expressed as riparian woodland P-loss prevention for erosion-derived material from the lateral zone adjacent to the stream (kg P and ha^−1^ [whole catchment] year^−1^).	P is used as proxy for top soil to avoid any possible double counting. Median low-end potential P loads for grassland and arable land (from Venohr et al. 2017) are reduced relative to the proportion of the river length that has riparian woodland. If this proportion is 1, all the potential load are retained. Grassland has 1 kg ha^−1^ year^−1^ available for erosion, cropland 2 kg ha^−1^ year^−1^. A low-end conservative value estimate for P is derived from an artificial fertilizer market price of 1.1322 € kg P^−1^ from a 2010 median market price at www.indexmundi.com
Flood prevention (2.2.1.3)	Damage function based on the risk of a 1/100 yr flood and a median distribution of different land use types over the river corridor (0–7 € ha^−1^ [catchment] year^−1^). It is assumed that no flood damage occurs in Lillebæk, as this first order stream directly discharges into the sea and only runs through agricultural land	Assumption is that one flooded upstream reach prevents the damage of flooding a median downstream reach of equivalent area. Value of built-up land is particularly high (252 € m^−2^, agricultural land has 7, and woodland has 1). This is adjusted to the height of the flood wave relative to property or crop (we use 0.2), and normalized to an annual value with a factor 1/100. Based on De Moel and Aerts (2011), and normalized to the whole catchment
Pest regulation (2.2.3.1 and 2.2.3.2)	Expressed as a modulation of crop productivity (provisioning service 1.1.1.1 above) linked to the presence of woodland and hedges as source of pest control. Modulation is a simple knowledge rule: if woodland cover < 25%, then crop productivity reduced to 80%	Based on Tscharntke et al. (2012)
Water quality improvement: nutrient retention (2.2.5.1)	Waterborne phosphorus retention in stream and in riparian floodplain during a flood	Only phosphorus is used to conservatively prevent double counting. From load reduction per stream km as well as P sedimentation during a flood event and combined with a conservative low market price for P of 1.1322 € kg P^−1^ derived from artificial fertilizers in the same way as for erosion control. Load reduction per km of stream length is derived from De Klein and Koelmans (2011), and Olde Venterink et al. (2003) at around 200 kg P km^−1^ river length for low land rivers and conservatively reduced to 10 kg P km^−1^ river length, because of a higher slope and flow in the current systems in accordance with unpublished MONERIS model estimates by Gericke & Venohr. P-load reduction during flood wave passage is estimated for Ovre Halden from P sedimentation during the flood and floodplain area. The two retention mechanisms are normalized to catchment area
Water temperature regulation through riparian shading (2.2.6.2)	Shading affects the probability of trout survival and is expressed as a modulating effect on the cultural service angling. Knowledge rule: if 50% of the main river length is shaded by woodland, then 100% survival, else a stepwise decline in survival to a residual survival of 10%.	The fish survival knowledge rule is directly linked to the value estimate due to recreative angling. Trout survival knowledge rule is based on Broadmeadow et al. (2011) who showed that in a stream in S England periods with water temperature over 25 °C were effectively prevented if woodland cover of the stream exceeded 50% of its length
Cultural services		
Recreative angling (taken separate from hunting, 1.1.6.1)	Angling days per km of stream	This is based on the proportion of households with one angler and the number of households in a catchment, and a low-end conservative estimate of their reported willingness to pay for angling per year from Arlinghaus (2004: 275 € year^−1^ per angler); value is also normalized to catchment area
Active recreation in the river and its floodplain corridor (all in CICES category 3.1 pooled)	Separate local estimates for the number of local and residents and tourist visitors that use and appreciate the area per year from local statistics. Multiplied with their willingness to pay for this and modulated by a knowledge rule on the appreciation of a scenic landscape: if forest cover declines below 20% tourist appreciation drops to 60%, if it is above 70% then appreciation drops to 80%.	Knowledge rule on scenic landscape is based on Frank et al. (2013); willingness to pay of residents and visitors based on Elsasser et al. (2010) and Boesch et al. (2018)
Biodiversity non-use (all in CICES category 3.2 pooled)	Number of households willing to pay for nature conservation	Based on a nationwide study in Germany (Boesch et al. 2018) but adjusted to local population sizes from municipality national statistics and then normalized to catchment area

### Land use change effects on ecosystem services provision

Obviously, deliberate and unplanned changes in land cover can have major direct as well as indirect impacts on how mankind benefits from the land. The Hubbard Brook experiment (Likens et al. 1978) is an iconic example of forest removal effects: stream discharge volume and variability, and suspended solids loss and nutrient exports increased. Similar changes may occur when Nordic forest cover and exploitation change, as occurred historically with changing land use (Meyer-Jacob et al. 2015). Intensified forestry has been shown to affect dissolved organic carbon mobilization, nutrient leaching, and dead wood prevalence (Schelker et al. 2012; Forsius et al. 2016). Changes in forest cover and exploitation, thus, may affect hydrology, carbon sequestration, and a range of regulating services, but also have consequences for cultural services such as the non-use existence value of biodiversity (Ranius and Roberge 2011) and recreative appreciation (Frank et al. 2013). Different effects could counteract or strengthen each other (trade-offs, bundles, or synergies, e.g., Raudsepp-Hearne et al. 2010; Martín-López et al. 2012; Queiroz et al. 2015). Among others Nelson et al. (2009) and Laudon et al. (2011) argue that such interactions can only be assessed well by integrating at the larger landscape scale. As one cannot gauge the strengths of possible interactions beforehand, we used multiple scenarios for the development of a future Nordic bio-economy. We use ‘catchment’ as our spatial object rather than ‘landscape’ or ‘ecosystem’ because it allows us to construct water balances which are important for the estimation of several provisioning and regulating services.

### Scenarios of a future Nordic bio-economy for the case study catchments

Rakovic et al. (2020) have articulated the Shared Socioeconomic Pathways (SSPs) from, among others, O’Neil et al. (2017) as narratives of plausible but contrasting trajectories for a developing bio-economy in Nordic societies including land use, and renamed these new articulations to Nordic Bio-economy Pathways (NBPs). We present short narratives for each (Table 2). Our time horizon is 2050. By that time the trajectories of geophysical climate change described by the different Representative Concentration Pathways (RCPs) will not yet be markedly different beyond the projected uncertainty bands (IPCC 2014), so we have chosen not to include these in our analysis. We selected two contrasting catchments: Lillebæk on eastern Fyn, Denmark, and Ovre Haldenvassdraget in southeastern Norway. The former is a small first-order catchment mainly used as cropland and directly draining into the Great Belt (latitude + longitude for the outflow: 55°06′ N, 10° 46 E), and the latter is a large, mainly forest-covered catchment draining through its lower part into the Skagerrak (59° 29′ N, 11°39′ E). These catchments are quite well studied (e.g., Hansen et al. 2013 and Greipsland 2015). Immerzeel et al. (submitted) have carried out in-depth choice experiment surveys among residents and visitors of Ovre Haldenvassdraget and Odense Å catchment, which is adjacent to Lillebæk. The surveys focused on preferences for nature conservation, water quality, landscape, land use type, and land use intensity. Based on the outcome of an elaborate stakeholder consultation in Norway on the plausibility and consistency of the NBPs, and an internal expert meeting on the same in Denmark, we have deduced the plausible changes in land use for each of the scenarios (see Supplementary Material S1).

**Table 2:** Brief narratives of the BIOWATER Nordic Bioeconomy Pathways (NBPs), by Rakovic et al. (2020) based on the SSPs from O’Neill et al. (2017)

NBP	Narrative
NBP1: Sustainability first—closing the loops	Societies around the world increasingly recognize the environmental, social, and economic costs of disconnected, resource-intensive production, and consumption patterns. The development thus shifts to a more sustainable path, which respects perceived environmental boundaries and places human well-being ahead of economic growth. The changes in energy systems are directed towards renewables and high resource efficiency, coupled with consideration of the environmental footprint from the cradle to the grave. Along with the low resource-intensive lifestyles, this leads to a low overall energy use. In the Nordic countries, the bioenergy share of energy use is relatively high and based on waste, residues and by-products. Policies in the bio-economy sector are oriented towards development of sustainable and circular supply chains. Coupled to this there is a shift from linear to more circular, regionally diverse, and resource efficient land use, which includes maintaining a balance between nutrient input and output. The widespread environmental awareness of societies leads to low meat and low dairy diets. In this sustainability-oriented world, there are low challenges to climate change mitigation and low challenges to adaptation to the effects of climate change
NBP2: Conventional first—do not rock the boat	This world follows typical recent historical patterns with uneven development and income growth. There is a concern for local pollutants but moderate success in policy implementation and slow progress in achieving the sustainable development goals. In the Nordic energy sector, some investments in renewable energy systems are made but society continues to rely on fossil fuels. The bioenergy share of energy use is relatively low although there are some investments in novel technology. Within the bio-economy sector, there is an overall weak focus on sustainability with continued dependence on disconnected (linear) supply chains from production of biomass to consumption. Although overall consumption is material-intensive, there is a slight downward trend in meat consumption. In this middle-of-the-road society there are moderate challenges to climate change mitigation and adaptation
NBP3: Self-sufficiency first—building walls	The world is characterized by rising regional rivalry driven by growing nationalistic forces and the Nordic countries have become allies in a fragmented Europe. International trade is strongly constrained and policies are oriented towards security, while there is low priority for environmental issues. The importance of developing the Nordic bio-economy therefore becomes a matter of regional security, placing self-sufficiency aims high up on the agenda. Energy consumption is high and prevailing Nordic energy systems and supplies are expanded, such as hydropower and Norwegian oil. There is also a moderate rising trend in domestic bioenergy production, including biofuels produced from mainly organic waste and forest harvesting residues. Technology development is, however, slow in all sectors. There is also a low priority for environmental considerations, consumption is material-intensive, and diets are meat rich. Due to lack of international cooperation and low environmental awareness, there are high challenges to climate change mitigation and adaptation
NBP4: City first—maintaining the divide	In a world with unequal investments in human development and rising differences in economic opportunity and political power, a gap widens across and within countries between a small affluent elite and underprivileged lower-income groups. Environmental policies are centered on local concerns with little attention to vulnerable areas or global issues. In the Nordic countries, segregation between societies in overlooked residential areas and more valued prosperous regions continues to lower societal cohesion. Rural areas that are not favorably situated for tourism are increasingly neglected because policy is oriented towards the benefit of those with economic power. Big corporations gradually take over the land-based bio-economy sector at the expense of small-scale family farms and forest owners. Due to an uncertain fossil fuel market, there are diversified investments in the energy sector, including efficiency and renewables. The bioenergy share of energy use follows an upward trend facilitated by rising import of bioresources to the Nordic countries. Due to some low carbon investments and a well-connected international political and business class there are low challenges to climate change mitigation. Challenges to adaptation to the effects of climate changes are, however, high
NBP5: Growth first—running on the treadmill	Spurred by high economic growth and rapid technological development, this society trusts in that competitive-markets, new technology, and investments in human capital is the path to sustainable development. Regarding environmental policy, there is a focus on local issues with obvious benefits to human well-being, whereas global issues receive little attention. In this society, lifestyles are material-intensive and diets are meant rich. The energy and resource intensity is high and there is a heavy reliance on fossil resources. With increasingly connected global markets, biomass production moves towards more large-scale and regionally specialized systems, also in the Nordic countries. There are, however, limited incentives to develop the bioenergy sector. In this fossil-fueled society, there are high challenges to climate change mitigation. However, a highly engineered infrastructure leads to low challenges to adaptation

## Results

**Fig. 2:**
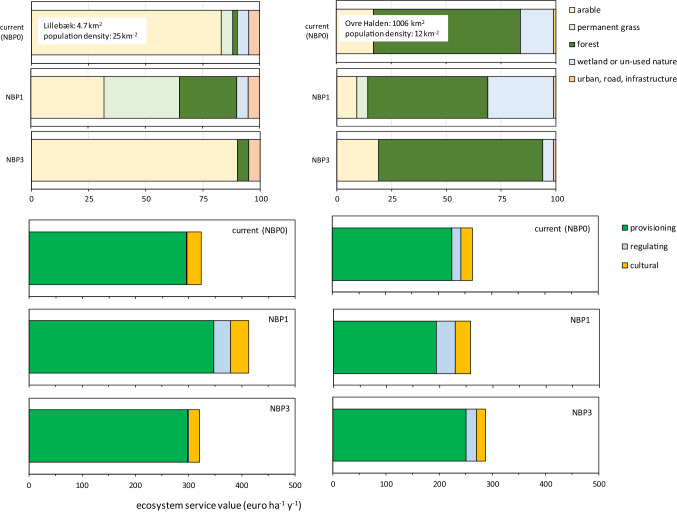
Land use distribution in the Lillebæk and Ovre Halden catchments for the scenarios NBP0, 1, and 3 (upper charts, units are % of total land cover), and consequent effect on estimated ecosystem service delivery (bottom charts). Land use types are the pooled CORINE classes indicated in Fig. 1. Only the effects of NBP0, 1, and 3 are shown because in Lillebæk NBP2 and 4 are similar to NBP0 and NBP5 is similar to NBP3; In Ovre Halden, NBP 2 is very similar to NBP0 and NBP4 and 5 are similar to NBP3. Estimation of ecosystem service benefit estimates is only estimated for NBP0 and NBP1, to illustrate the potential of the method. Ecosystem services are aggregated into provisioning, regulating, and cultural services and summed to estimate an approximate total economic value (TEV)

Differences in projected land use distribution among most scenarios for the two catchments were comparatively limited (Fig. 2, upper panel, only NBP0, 1, and 3 are shown), and only the most circular and sustainability-oriented scenario NBP1 led to a substantial redistribution of land use towards 2050, in both catchments.

In Lillebæk, an agricultural diversification appears to lead to an increase in net agricultural farmgate revenue (Fig. 2 lower panel), because dairy is estimated to be more profitable than cereal production (Table 2) based on the agricultural statistics over the last decade across Europe (e.g., Mueller and Mueller 2017). It must be noted that we have not included a higher market price for ecological or locally branded products as may have been plausible under NBP1. In addition, increased riparian woodland and wetlands under NBP1 contribute to a higher carbon sequestration and nutrient retention in this first-order catchment. Cultural services appear not to be affected in this small, largely agricultural catchment. The summed estimate of total economic value in Lillebæk is higher for NBP1 than for NBP0. In the Norwegian catchment, however, the scope for large-scale changes appears less, and when we assume a similar adjustment in agriculture towards a richer mixture of different farming systems in NBP1, still most of the catchment will remain forest-covered, and forestry products continue to dominate the provisioning services (two-thirds, or 110 of 193 € ha^−1^ [catchment] year^−1^). Here, provisioning services decrease in NBP1 due to this decline in forest area, whereas regulating services increase (from 17 to 35 € ha^−1^ [catchment] year^−1^), due to increased flood prevention in the larger area of wetlands and an assumed higher (shadow) market price for sequestered carbon. The summed TEV estimate is lower for NBP1 than for NBP0, as it is dominated by our estimate of forest net present value. In contrast to NBP1, the other scenarios led to limited changes in land use and ecosystem service delivery, and we therefore have chosen to only show NBP3 (see explanation in caption of Fig. 2).

To consider significance of these differences, we use a relative error from an earlier study using a similar method (Vermaat et al. 2016: relative standard error 0.24 for TEV across 16 cases). Only the relative difference between the regulating services of NBP0 and NBP1 is much higher than this standard error in both catchments (53 and 2.6 times for Lillebæk and Ovre Halden, respectively). These are thus likely significant; the others are not, so our illustration suggests that NBP1 likely increases regulating services and herewith TEV.

## Discussion

Based on the two cases, it appears that only a comparatively ‘extreme’ scenario leads to major changes in land use with subsequent effects on the pattern in ecosystem delivery. However, it must be borne in mind that we have not included any effect of land use intensity. Thus, one can expect major effects of, for example, a more intense biomass-harvesting-oriented forest management on regulating services such as nutrient retention and carbon sequestration in Ovre Halden and similar catchments with a predominance of forest (cf. Laudon et al. 2011), or of a prolonged and systematically reduced focus on environmental protection, but we have not modeled that. The former appears plausible in NBP3, the latter in NBP3, 4, and 5 (Table 1). Including management intensity in our modeling would thus likely lead to more pronounced differences among NBPs. One could also foresee an effect on recreational benefits as citizens were found to strongly appreciate small-scale, low-intensity forest management practices (Juutinen et al. 2014). Our findings from two strongly contrasting catchments also suggest that national policy and the overall landscape of a catchment together may define the development of a scenario trajectory and the importance of different services, in line with Queiroz et al. (2015), who found clear gradients in ecosystem service trade-offs moving from near-capital peri-urban landscapes to the remote rural periphery in Sweden.

Do we meet the six requirements (*italicized below*) we set for the analytical framework? First, the requirement of *minimal assumptions* is met rather well. The ‘Mononen-cascade’ we apply here has three major underlying assumptions: (1) that a land cover classification is capable of sufficiently grasping ecosystem structure and its variation across landscapes; (2) the CICES classification of ecosystem service flows and particularly our selection from this exhaustive list covers all potentially relevant services; and (3) final monetary value estimates are acceptable. This compilation of value estimates of societal benefits with several highly different underlying approaches and assumptions is probably the most frequently disputed aspect of TEV estimates (a.o. Schröter et al. 2014) but it allows aggregation and comparison across scenarios and services, and thus can be used to inform policy. We argued above that it can be considered a form of weighing as in MCA.

Second, we are convinced that our use of the CORINE land use system is highly *traceable* as it is harmonized and well tested across Europe. Third, we show that we can track our different services in a *transparent* way (Fig. 1) and we have experienced that the cascade form has dictated consistency in our formulation of the different services (cf. Table 1). Fourth, our spreadsheet application for the two cases shows that *scenario application* is possible, without intervention of a complex modeling environment (Sharps et al. 2018). Fifth, we have not *disaggregated* our catchments *spatially*, but the framework can easily be populated for different sub-catchments, of which the parallel estimates can be aggregated, or be linked to a catchment model which has routed flows or hierarchical nesting (cf. Nelson et al. 2009). Our approach can be adjusted easily to a spatial script to obtain *spatial explicitness* because it is using CORINE classes. Including spatial detail allows addressing location effects of, e.g., recreational opportunities, erosion prevention linked to slopes, water and nutrient retention in wetlands, biodiversity hotspots for conservation, and downstream functions of the drainage network. Sixth, the quantification of the different services we have developed (Table 1) has been necessarily kept simple, and together with the spreadsheet form and graphical output this will greatly facilitate *stakeholder interactions*.

We have not included uncertainty and variability in the current spreadsheet application other than by implementing contrasting scenarios, but this can be realized by adding more cases, by coupling to a routed catchment model that has been calibrated and validated on existing data, or with an ex-post Monte Carlo assessment. Also, we have not included an analysis of possible effects of these changes on different groups of beneficiaries, which are likely not benefitting equally, and this would be highly relevant to complement the information provided in stakeholder interactions. Furthermore, we limited the articulation of our scenarios to land use change and have not included any effect of possible changes in demography, behavior, or economic strength that would be consistent with these scenarios.

An important point of criticism arguably is the variety of methods we use to arrive at our estimates of monetary value (see e.g., Bateman et al. 2011). Provisioning services have generally been based on net farmgate revenues in a market setting, regulating services on avoided costs, and cultural services on stated preference. These different estimation methods have different uncertainties. For instance, our use of transferring value estimates from one study site to another contains inherent uncertainty. The estimates used for crops, dairy, timber as well as recreation and non-use values in this paper, all contain a form of benefit transfer, though not based on transfer from a single study site. Thus, we assume that beneficiaries across Europe value ecosystem services in the same way, which might not be the case (Riera et al. 2012). For the aim of the present analysis, i.e., the development of the Mononen-cascade and a test with scenarios, the use of benefit transfer is not a fundamental flaw, as we use the findings only in a comparative way.

Our scenario articulations for the development of Scandinavian economies towards a more circular bio-economy in 2050 were limited to the effects of changes in land use, and therefore did not pick up consequences of changes in land use intensity. Nevertheless, we found distinct effects on ecosystem service delivery, particularly in the truly sustainability-oriented scenario NBP1, where drastic changes in land use also led to changes in the pattern of ecosystem service delivery. The outcomes were markedly different in our two catchments. In the small Danish catchment, presently used mostly as intensive cropland, a more diverse land use distribution led to an overall increase in provisioning and regulating services, and an almost 25% higher estimated total economic value. In the larger, forest-dominated Norwegian catchment, however, a similar but less drastic diversification with forest still dominant led to an increase in regulating services at the expense of provisioning services, largely timber. Hence, here we observe a trade-off among services. A first tentative answer to the question how a future bio-economy would affect ecosystem service delivery in these Nordic catchments would thus be that this depends on how large the changes in land use-type distribution will be, on how land use intensity will change, and on how agricultural, environmental, and energy policies will be implemented. This will be the subject of a more comprehensive analysis in a larger number of catchments that we are developing from this current study. A second answer would be that NBP1 is likely closest of our five scenarios to what one intuitively imagines as a successful green bio-economy. Hence that would mean more diverse land use and a more diverse suite of ecosystem services provided, with a stronger focus on regulating services, possibly at the expense of a provisioning service, like timber production.

Our purpose was to explore the possibilities and limitations of our ‘Mononen-cascade’ approach. Clear limitations are the absence of spatial dependencies and disaggregation due to the simple spreadsheet structure, and the nearly absent treatment of uncertainty. The same simple and graphical structure, however, also opens up for exploration and hence is highly amenable for stakeholder interactions. In short, we therefore argue that the framework we have developed sufficiently meets our requirements, and we advocate for a wider application, also for cases where data availability is limited (Vrebos et al. 2015).

## Conclusion

If the transition of Nordic societies towards a bio-economy would include a focus on sustainability and environmental protection, this would likely lead to an overall increase in and a more varied range of ecosystem services delivered. If, however, a bio-economy would largely focus on increased biomass outtake, ecosystem service delivery would most likely not be increased. The impacts of such a bio-economic development, however, appear catchment-specific, and this poses a challenge for policy implementation. Our proposed ecosystem service assessment using the ‘Mononen-cascade’ may prove useful in addressing such challenges by identifying the impacts on ecosystem services and associated trade-offs at a catchment scale.

## Electronic supplementary material

Below is the link to the electronic supplementary material.

Excel file with land us projections and estimates of ecosystem service delivery. Supplementary material 1 (XLSX 4639 kb)
